# Novel Prognostic Model Based on Immune Signature for Head and Neck Squamous Cell Carcinoma

**DOI:** 10.1155/2020/4725314

**Published:** 2020-10-19

**Authors:** Yingying Wang, Yu Xu, Qingquan Hua, Yang Jiang, Peiqiang Liu, Wei Zhang, Rong Xiang

**Affiliations:** ^1^Department of Otolaryngology-Head and Neck Surgery, Renmin Hospital of Wuhan University, Wuhan, China; ^2^Research Institute of Otolaryngology–Head and Neck Surgery, Renmin Hospital of Wuhan University, Wuhan, China

## Abstract

**Background:**

Deciphering the immune characteristics within tumors and identifying the immune signals related to the prognostic factor are helpful for the treatment and management of tumor patients. However, systematic analysis of immune signatures in head and neck squamous cell carcinoma (HNSCC) remains largely unstudied.

**Methods:**

A total of 718 immune-related genes were extracted from RNA sequencing data from 519 HNSCC patients in the TCGA database, and survival analysis with integrated bioinformatics analyses was performed to build the final predictive prognosis model.

**Results:**

The 178 survival-associated genes (*P* < 0.05) participated in important immune functions, including immune cell activation and migration. Multivariate regression analysis using 93 genes (*P* < 0.01), together with survival-associated clinicopathological parameters, identified 35 independent prognostic factors. The most significant 8 independent factors were CD3E, CD40LG, TNFRSF4, CD3G, CD5, ITGA2B, ABCB1, and TNFRSF13b. The final prognostic model achieved outstanding predictive efficiency with the highest AUC of 0.963.

**Conclusion:**

Our prognostic model based on the immune signature could effectively predict the prognosis of HNSCC patients, providing novel predictive biomarkers and potential therapeutic targets for HNSCC patients.

## 1. Introduction

Head and neck squamous cell carcinoma (HNSCC), including tumors generated in the oral cavity, oropharynx, larynx, or hypopharynx, is the sixth most frequently diagnosed cancer worldwide, with only a 40–50% 5-year survival rate [1]. Despite considerable advancements in recent years in imaging techniques, surgery, radiotherapy, and chemotherapy, the survival rate in patients with HNSCC has not improved greatly and remains unsatisfactory [2–4]. Even worse, it was reported that the HNSCC mortality rate has increased from 2012 to 2016 for tumors associated with the human papillomavirus (HPV).

HNSCC is a heterogeneous disease with unsolved complexity in terms of etiology, pathogenesis, morphological characteristics, clinical features, and natural history. So far, the most critical risk factors are tobacco use and heavy alcohol consumption [5–7]. The prognosis of patients with HNSCC is mainly indicated by the stage at diagnosis [1]. Early stage cancers are treated with radiotherapy or surgery and have a relatively better prognosis than advanced HNSCC patients. Studies have shown that the response rate of single-modality therapy (either surgery or radiation alone) in early stage tumors was significantly higher than that of advanced tumors. Disappointingly, the majority of patients were diagnosed at advanced stages with distant metastases. Therefore, a novel prediction model for the survival of HNSCC was greatly needed.

With limited understanding of the genetic and biological heterogeneity of the HNSCC, few effective therapeutic strategies were available compared with those for other tumor types. The exploration of the TP53/RB pathway, PI3K/AKT/MTOR pathway, EGFR pathway, and other signaling pathways brought novel insights into the initiation and development of cancer and resulted in several successful targeted therapeutics. From the representative targeted therapy (EGFR-tyrosine kinase inhibitor, EGFR-TKI) to revolutionary immunotherapy, these therapeutics demonstrated that a better understanding of signaling pathways in cancer might benefit the development of novel therapeutics. Furthermore, illumination of the molecular factors involved in the prognosis of HNSCC not only guides the treatment of HNSCC patients in a clinical way but also helps the exploration of novel targeted therapies or combined modality treatment strategies [8].

Currently, the application and mechanisms of immunotherapy are being extensively explored in HNSCC and other malignancies; immune signatures were reported to be reliable prognosis predictors for other types of cancer. However, the potential role of immune-related genes in HNSCC remained largely unstudied, and there is no effective prediction model for HNSCC patients. The purpose of this study was to investigate the potential utility of the immune-related genes in the prognosis of HNSCC patients. This study systematically analyzed the prognostic value of 718 immune-related genes in 519 HNSCC patients and constructed a powerful prognostic prediction model for HNSCC patients.

## 2. Materials and Methods

### 2.1. Patient Cohort and Data Curation

The RNA sequencing data of tumor tissues from 519 HNSCC patients were downloaded from The Cancer Genome Atlas (TCGA) data portal (https://cancergenome.nil.gov/) with matching clinical information. In total, 718 immune-related genes curated from the nCounter® PanCancer Immune Profiling Panel (NanoString) were extracted from the RNA sequencing data.

### 2.2. Survival Analysis of the Immune Signatures in HNSCC

Overall, 509 HNSCC samples, which were fully characterized with at least 30 days of overall survival (OS), were included in the survival analysis performed using the survival package (version 3.1-7) in R (version 3.3.1). Patients were divided into high and low groups by median cut based on the expression of each gene, followed by univariate COX regression analysis and log-rank test to identify immune-related genes with prognostic ability. Then, Kaplan-Meier curves were drawn to compare the survival rates between the two groups. Together with clinical parameters, multivariate COX regression analysis was used to remove unnecessary factors and to build the prediction model for the overall survival of HNSCC patients. To compare the efficiency of the prognostic predictors, the survivalROC package (version 1.0.3), which allows for time-dependent ROC curve estimation with censored data, was used to estimate the area under the curve (AUC) of the receiver-operator characteristic (ROC) curve for each factor.

### 2.3. GO and KEGG Analysis

Gene Ontology (GO) and Kyoto Encyclopedia of Genes and Genomes (KEGG) enrichment were analyzed by GeneMANIA (Version 3.4.1), and 178 survival-associated genes (*P* < 0.05) from the univariate COX regression were used to perform GO and KEGG analysis.

### 2.4. Statistical Analysis

The log-rank test was used to analyze the difference in OS, and the hazard ratio (HR) was determined from a Cox proportional hazard model using the survival package (version 3.1-7). Survival models were compared with the survivalROC package (version 1.0.3), and Kaplan-Meier plots were used to visualize survival differences. All tests were two-sided, and a *P* value less than 0.05 was considered statistically significant unless stated otherwise. All statistical analyses were performed in R package (version3.3.1).

## 3. Results

### 3.1. Study Design and Analysis Pipeline

**Figure 1:**
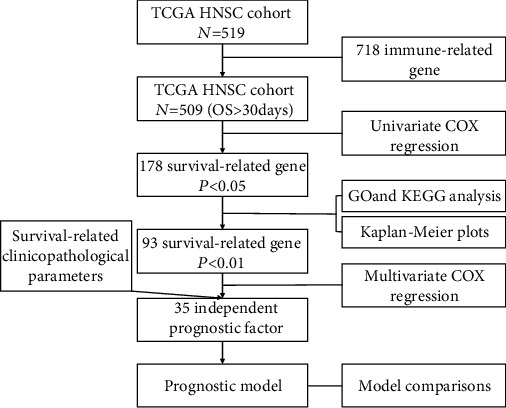
Workflow demonstrating the establishment and validation of a prognostic predictive model in HNSCC. The signature consists of 35 immune-related genes in HNSCC, which was established and validated using expression data from the TCGA database (training dataset).

**Figure 2:**
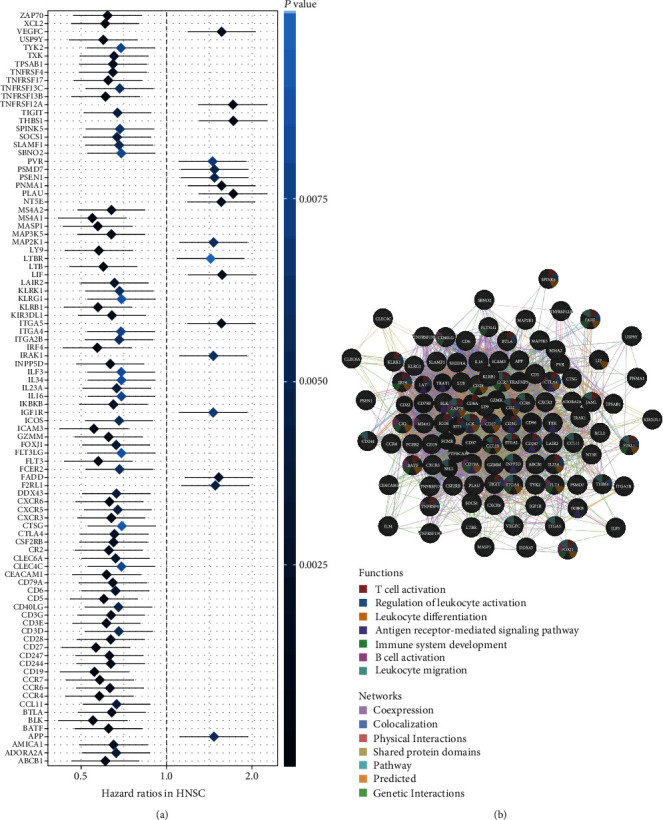
Prognostic immune signatures in HNSCC and enriched GO and KEGG analysis. (a) Ninety-three genes with prognostic ability were screen out by the univariate Cox analysis and log-rank test for TCGA HNSCC datasets (*P* < 0.05). Seventy-five genes (HR < 1) were favorite prognosis factors, while 18 genes (HR > 1) were adverse prognosis factors in HNSCC. (b) Significantly enriched GO and KEGG were analyzed. Function and networks of these dysregulated genes are presented.

**Figure 3:**
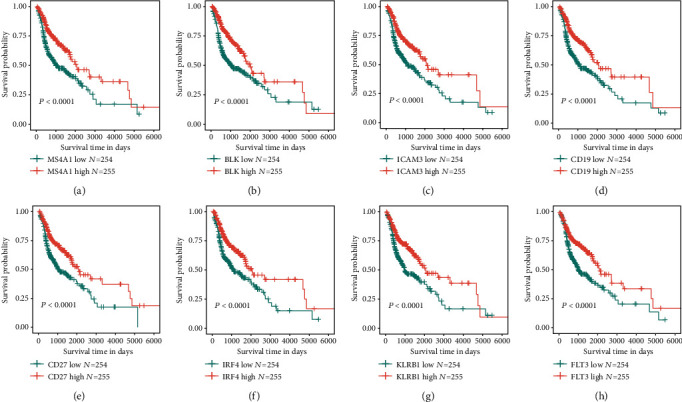
Kaplan-Meier plots of the top eight survival-related genes in the HNSCC datasets.

The overview of the study design and analysis pipeline is shown in Figure 1. Univariate Cox regression analysis of 718 immune-related genes from 519 HNSCC patients in TCGA database was performed. There were 178 genes (*P* < 0.05, Supplementary Figure 1) and 93 genes (*P* < 0.01, Figure 2) that significantly correlated with OS in the univariate analysis, respectively. Then, GO and KEGG analysis was used to demonstrate the potential role of these genes in essential pathways in immune responses. Kaplan-Meier plots of the top 8 survival-associated genes are shown in Figure 3. Relationships between clinical parameters and outcomes of HNSCC patients were also tested to verify the effectiveness of the survival data from TCGA cohort. Next, a multivariate Cox regression analysis was performed to establish the prognostic model with the candidate immune-related genes and clinicopathological parameters. Finally, ROC curves were applied to compare the efficiency of these predictive models and genes.

### 3.2. Characteristics of the Patient Cohort

Among the 519 HNSCC patients, a total of 509 HNSCC patients with OS > 30 days were enrolled in this study with detailed clinicopathological information provided by the TCGA database. The age at diagnosis ranged from 19 to 89 years old (median: 61 years), and 383 patients were male (73.8%). The median follow-up time was 855 days, ranging from 11 to 5,480 days, and 220 patients died by the end of follow-up.

### 3.3. Immune Signatures Associated with OS in HNSCC

To explore the potential role of immune signatures in HNSCC patients, specifically whether there are significant associations between gene expression and prognosis of HNSCC patients, expressions of 718 immune-related genes were extracted from RNA-sequencing data from HNSCC patents. Survival analysis by univariate Cox regression analysis using the survival package (Version 2.41.3) in R was performed with the HNSCC cohort of 509 patients for each gene. The results showed 178 genes (*P* < 0.05) and 93 genes (*P* < 0.01) that were significantly associated with the overall survival of HNSCC patients. The detailed hazard ratios (HRs) and *P* values are listed in Supplementary Figure 1 and Figure 2(a). High expressions of 139 genes (*P* < 0.05) and 75 genes (*P* < 0.01) were significantly associated with better overall survival, whereas 38 genes (*P* < 0.05) and 18 genes (*P* < 0.01) were associated with adverse prognosis in HNSCC (Supplementary Figure 1 and Figure 2(a)). GO annotation and KEGG pathway enrichment analysis were conducted among the 178 survival-associated immune genes to understand the underlying functions of these immune signatures in HNSCC. As a result, these 178 survival-associated genes participated in critical immune functions, such as the participation of MS4A1, BLK, CD19, and CD27 in B cell activation; ICAM3 in the regulation of leukocyte activation and leukocyte migration; and IRF4, CD5, and CD3E in T cell activation. Other functions include leukocyte differentiation, antigen receptor-mediated signaling pathway, and immune system development (Figure 2(b)).

Among 178 survival-associated genes, the top eight most significant genes were MS4A1, BLK, ICAM3, CD19, CD27, IRF4, KLRB1, and FLT3 (Figure 3). Kaplan-Meier curves are presented in Figures 3(a)–3(h), and *P* values by log-rank tests are provided. The hazard ratios (HRs) were MS4A1 (HR = 0.548, 99% CI: 0.415–0.721) (Figure 3(a)), BLK (HR = 0.550, 99% CI: 0.416–0.726) (Figure 3(b)), ICAM3 (HR = 0.554, 99% CI: 0.420–0.731) (Figure 3(c)), CD19 (HR = 0.557, 99% CI: 0.422–0.735) (Figure 3(d)), CD27 (HR = 0.564, 99% CI: 0.428–0.742) (Figure 3(e)), IRF4 (HR = 0.570, 99% CI: 0.432–0.750) (Figure 3(f)), KLRB1 (HR = 0.573, 99% CI: 0.435–0.753) (Figure 3(g)), and FLT3 (HR = 0.575, 99% CI: 0.436–0.756) (Figure 3(h)), respectively.

### 3.4. Internal Validation of Survival-Associated Clinical Parameters

**Figure 4:**
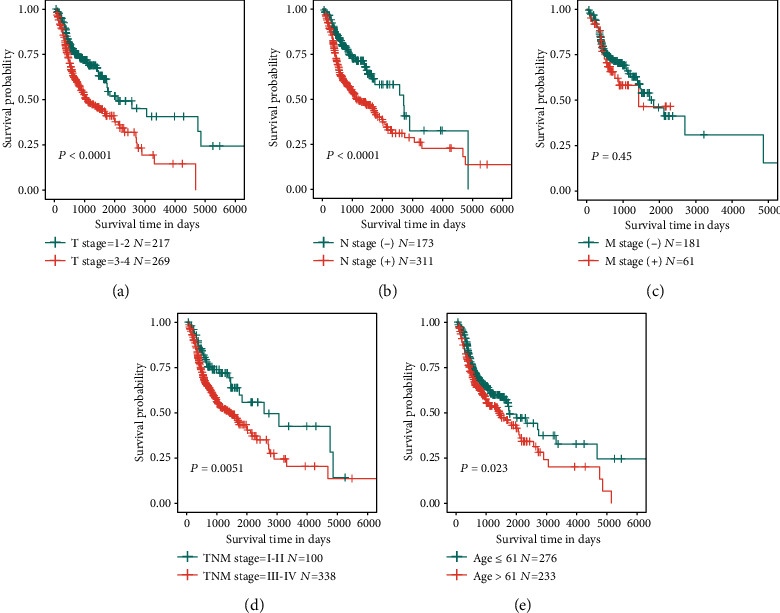
Clinical parameters were significantly associated with OS in HNSCC patients. Kaplan-Meier survival curves indicating survival probability are shown according to diverse clinical factors: (a) T stage, (b) N stage, (c) M stage, (d) TNM stage, and (e) age.

Before multivariate Cox regression analysis, survival analyses were also performed among important clinicopathological parameters with patients divided into two groups. Compared with patients in the low-risk group, high-risk group patients had a worse OS. In TCGA HNSCC cohort, patients in groups for T stages 3–4 (Figure 4(a)), N stage (+) (Figure 4(b)), M stage (+) (Figure 4(c)), TNM stages III–IV (Figure 4(d)), and age > 61 (Figure 4(e)) had significantly shorter OS (*P* < 0.05). The corresponding Kaplan-Meier plots are shown in Figures 4(a)–4(e), highlighting the prognostic value of clinicopathological factors, indicating that the data were verified to be reasonable and effective.

### 3.5. Multivariate Analysis and Model Construction for HNSCC Patients

To determine the independent factors for prediction of overall survival, clinicopathological parameters, including T stage, N stage, M stage, TNM stage, and age, which were significantly associated with OS as mentioned above (Figures 4(a)–4(e)), were selected to be included in the multivariate COX regression analysis.

**Figure 5:**
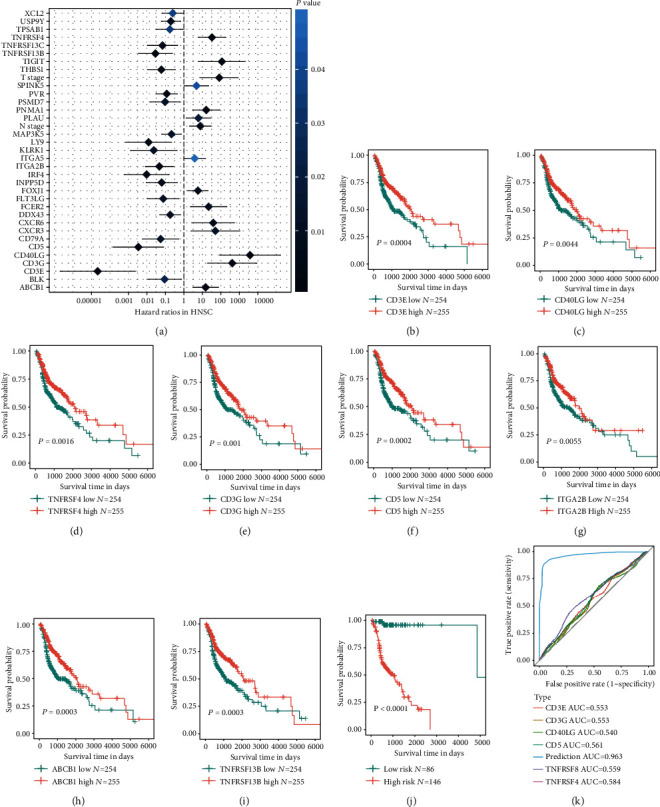
Immune signatures with multivariate Cox regression with prognostic capabilities in HNSCC and enriched GO and KEGG. (a) Thirty-five genes with prognostic ability were screen out by univariate Cox analysis and log-rank test for TCGA HNSCC datasets (*P* < 0.05). Twenty genes with hazard ratios (HR) < 1 were favorable prognosis factors, while 15 genes with HR > 1 were adverse prognosis factors in HNSCC. (b–i) Kaplan-Meier plots of the top eight independent survival-related genes. (j–k) Kaplan-Meier curves and time-dependent ROC curves of the prediction model.

Then, multivariate COX regression analysis was performed in these potential survival associated genes (*P* < 0.01) along with the clinicopathological parameters to construct a prognostic model. Results showed that only T stage (HR = 81.497, 95% CI: 7.790–852.496, *P* = 0.00024) and N stage (HR = 7.677, 95% CI: 2.020–29.177, *P* = 0.0028) could act as independent predictors for patients' OS. A total of 35 factors, including T stage and N stage, were significantly associated with overall survival (*P* < 0.05) which were latent independent factors in HNSCC. Among these potential independent factors, 20 factors were favorable genes (HR < 1), whereas 15 factors (HR > 1) were adverse prognosis predictors (Figure 5(a)). The top 8 independent survival genes were CD3E, CD40LG, TNFRSF4, CD3G, CD5, ITGA2B, ABCB1, and TNFRSF13b. The Kaplan-Meier plots in Figures 5(b)–5(i) display that all these 8 genes correlated with patient prognosis significantly. Among these 8 genes, high expression of CD5, ITGA2B, and TNFRSF13B indicated a favorable prognosis, while high expression of CD3E, CD40LG, TNFRSF4, CD3G, and ABCB1 were poor prognostic factors for HNSCC. Therefore, we built the prediction model for the overall survival of HNSCC patients with 35 factors, including clinicopathological parameters. Surprisingly, the prediction model demonstrated powerful efficiency in predicting good or poor overall survival of HNSCC patients as displayed in Figures 5(j) and 5(k). ROC curves by the survivalROC package in R showed that the prediction model was much better than each individual parameter. The AUC of the prediction model was 0.963, which was much greater than other genes or clinical parameters, suggesting that our model can effectively predict the prognosis of HNSCC.

## 4. Discussion

Over the many years of in-depth studies in the field of complicated and interrelated immune responses of cellular and molecular mechanisms, immunotherapy has become an effective way to treat cancer. The treatment of cancer is undergoing a fundamental transformation, and its traditional treatment methods are being overturned by immunotherapy. More and more immunotherapies have been used in cancer therapy alone or in combination, and many clinical trials are testing the efficacy of immunotherapy. However, there are still many difficulties due to the lack of understanding of immunosuppressive sites and the pattern of immune cell functions. Understanding the immune characteristics of tumors and finding the immune signature related to tumor prognosis are conducive to early diagnosis and evaluation of treatment response and prognosis through immune biomarkers and noninvasive monitoring. Personalized immunotherapy based on individual genetic, molecular, and immune analysis is a possible goal in the future [9].

Increasingly, research has involved immunological studies of head and neck tumors. Although the immune research of head and neck tumors is still in its infancy, it is one of the most promising fields. However, due to the complex interactions between the host immune system and cancer, further research and development of key immune signatures affecting the prognosis of cancer are necessary to optimize future immunotherapies. In fact, accurate prognosis, therapeutic strategy, and identification of oncogenes were encumbered by unexpected heterogeneity in HNSCC. This complex heterogeneity may signify the difficulty to accurately predict patient outcomes in tumors that have not yet metastasized, due to the resistance of certain normal-looking cells to targeted therapies or their ability to generate and promote metastasis [10]. The main purpose of immunotherapy is to eradicate cancer cells by increasing the activity of the immune system [11].

Recently, tremendous advances in cancer therapy have been achieved using immune checkpoint inhibitors [12–18]. These studies highlighted the importance of the exploration of immune signatures. Furthermore, intense investigations into immunotherapies are also underway in HNSCC. Promising results from clinical trials showed that immunotherapy for HNSCC might be superior to the standard chemotherapy.

A total of 45 relevant clinical trials of immune checkpoint inhibitors have been performed since 2010. Pembrolizumab extended the duration of response in recurrent and/or metastatic (R/M) HNSCC by approximately 53 weeks. Therefore, pembrolizumab had obtained accelerated approval by the FDA for the treatment of refractory R/M HNSCC after platinum-based chemotherapy [19–26]. So far, two immunotherapeutic agents, nivolumab and pembrolizumab, were approved by the US Food and Drug Administration (FDA) for the management of platinum-treated refractory R/M HNSCC patients in 2016. In 2019, pembrolizumab was approved by the FDA for first-line care in patients with unanticipated R/M HNSCC [27].

The commonly used immunosuppressive checkpoint inhibitors in these studies included PD-1, CD27, and CTLA-4 [28], which were consistent with our study. In the univariate analysis, CD27 (HR = 0.564, *P* < 0.01), CTLA-4 (HR = 0.655, *P* = 0.002), and ENTPD1 (HR = 0.707, *P* = 0.012) were all significantly correlated with prognosis.

The construction of the gene prediction model has been applied in many tumors, such as esophageal cancer, lung cancer, and thyroid cancer, but there are only few relevant studies on head and neck tumors. In some studies, by comparing three separate comprehensive gene expression omnibus (GEO) databases, researchers claimed that overlapping differentially expressed genes [29] like SPP1, POSTN, and COL1A2 could be used as potential diagnostic indicators of head and neck carcinoma. However, classical factors of head and neck tumors were not taken into account in the study. Moreover, reports [30] showed that different methylation status by the MethylMix R package based on the *β* mixture model constructed using six genes (INA, LINC01354, TSPYL4, MAGEB2, EPHX3, and ZNF134) could predict OS in HNSCC patients. However, the area under the curve (AUC) for the model was only 0.723. Further, four candidate genes (TPM1, CLASRP, PRRC1, and DNASE1L1) were screened out among the 42,849 alternating splicing events identified in 10,121 genes in another study, which built a prognostic prediction model with an AUC of 0.704 [31]. These results were much lower than the AUC of 0.963 in our study.

In summary, our report systematically investigated the potential role of 718 immune-related genes in the OS of HNSCC patients. Dynamic involvement of immune signatures was identified by survival analyses, and a total of 35 factors remained as independent prognosis factors. Surprisingly, the final prediction model yields high efficiency in distinguishing good or poor OS in HNSCC patients. With future validations in larger HNSCC cohort and mechanism studies of these survival-related immune signatures, these novel predictors and therapeutic targets might assist in the management and immunotherapeutic treatment of HNSCC patients.

## Data Availability

The datasets used during the present study are available from the corresponding author upon reasonable request.

## Supplementary Materials

Supplementary Materials Supplementary Figure 1: 178 genes with prognostic ability was screen out by the univariate Cox analysis and log-rank test for TCGA HNSC datasets (*P* < 0.01). 139 genes (HR<1) were favorite prognosis factors, while 38 genes (HR>1) were adverse prognosis factors in HNSC.
